# Paclitaxel-induced neuropathy: potential association of *MAPT* and *GSK3B* genotypes

**DOI:** 10.1186/1471-2407-14-993

**Published:** 2014-12-22

**Authors:** Susanna B Park, John B Kwok, Clement T Loy, Michael L Friedlander, Cindy S-Y Lin, Arun V Krishnan, Craig R Lewis, Matthew C Kiernan

**Affiliations:** Neuroscience Research Australia, University of New South Wales, Kragujevac, New South Wales Australia; Prince of Wales Clinical School, University of New South Wales, Kragujevac, New South Wales Australia; School of Medical Sciences, University of New South Wales, Kragujevac, New South Wales Australia; School of Public Health, University of Sydney, Sydney, Australia; Department of Medical Oncology, Prince of Wales Hospital, Kragujevac, New South Wales Australia; Brain and Mind Research Institute, University of Sydney, Sydney, Australia

**Keywords:** Paclitaxel, Neuropathy, Neurotoxicity, MAPT, GSK3β

## Abstract

**Background:**

Paclitaxel treatment produces dose-limiting peripheral neurotoxicity, which adversely affects treatment and long-term outcomes. In the present study, the contribution of genetic polymorphisms to paclitaxel-induced neurotoxicity were assessed in 21 patients, focusing on polymorphisms involved in the tau-microtubule pathway, an important target of paclitaxel involved in neurotoxicity development.

**Methods:**

Polymorphisms in the microtubule-associated protein tau (*MAPT*) gene (haplotype 1 and rs242557 polymorphism) and the glycogen synthase kinase-3β (*GSK3β*) gene (rs6438552 polymorphism) were investigated. Neurotoxicity was assessed using neuropathy grading scales, neurophysiological studies and patient questionnaires.

**Results:**

A significant relationship between the *GSK-3B* rs6438552 polymorphism and paclitaxel-induced neurotoxicity was evident.

**Conclusions:**

Polymorphisms in tau-associated genes may contribute to the development of paclitaxel-induced neurotoxicity, although larger series will be necessary to confirm these findings.

## Background

The major cytotoxic target of paclitaxel is the microtubule system, which provides stability for cellular shape, signaling and mitosis. Paclitaxel binds to the microtubule component β-tubulin, interfering with microtubule dynamics and leading to microtubule stabilization, mitotic arrest and ultimately apoptosis in chemo-sensitive cancer cells [1].

Microtubules are also critical for axonal function and provide the major transport route for essential organelles to distal nerve endings [2]. Disruption of axonal transport may interrupt energy mechanisms, leading to axonal degeneration and neuropathy. Paclitaxel causes neuropathy as a prominent dose-limiting side effect [3] and induces microtubule aggregation in the peripheral nervous system [4] and interruptions in anterograde axonal transport [5], suggesting that microtubule dysfunction may be important in the development of neuropathy.

The microtubule-associated protein tau gene (*MAPT*) encodes the protein Tau, involved in tubulin assembly and polymerization [6]. *MAPT* has two major haplotypes, H1 and H2, which affect Tau splicing and expression [7]. The H1 haplotype is associated with increased *MAPT* transcription and tau expression [6, 8]. *MAPT* expression is also a marker of paclitaxel resistance, with low expression linked to improved treatment response [9]. Tau is phosphorylated by glycogen synthase kinase-3β (GSK3β), a signaling protein [10], which has also been linked to paclitaxel chemoresistance [11]. In addition, tau concentration may influence paclitaxel binding affinity to microtubules [12] and paclitaxel and tau may compete for the same β-tubulin binding sites [13].

Given the putative role of microtubule dysfunction in neurotoxicity, we investigated effects of genetic variation in *MAPT* and *GSK3B* on neurotoxicity in paclitaxel-treated patients.

## Methods

### Study design and patients

Paclitaxel-treated patients were referred by the Department of Medical Oncology, Prince of Wales Hospital. The study was approved by the South Eastern Sydney Area Health Service and University of New South Wales Human Research Ethics Committee. Participants provided written informed consent. Patients were excluded if there was another potential cause for neuropathy such as diabetes, or evidence of neuropathy (based on clinical examination or neurophysiological study) prior to chemotherapy treatment.

### Neurological assessment

Patients were assessed using the National Cancer Institute Common Toxicity Criteria for Adverse Events–neurosensory subscale (NCI). 18 patients underwent more specialized clinical neurological assessment, using the Total Neuropathy Scale-clinical version (TNSc) [14] and encompassing symptoms, pinprick and vibration sensibility, strength and deep tendon reflexes (total score 0–28). Patient neurotoxicity questionnaires (EORTC-CIPN20) were undertaken. Nerve conduction studies were undertaken to assess compound sensory action potential (CSAP) amplitude of the sural and median nerves. Stimulus–response curves were recorded from the sensory median nerve at the second digit and the current required to produce a CSAP of 90% maximal amplitude (i90) was determined using the QTracS stimulus delivery program (Institute of Neurology, UCL). i90 at the 4th week of paclitaxel treatment has been identified as predictive of neurological outcome [15].

### Genotype analysis

Lymphocyte-derived patient DNA samples were genotyped for MAPT Haplotype (H1 Haplotype), the single nucleotide polymorphism rs242557 in *MAPT*, and the single nucleotide polymorphism rs6438552 in *GSK3B*. Genotypes were coded in three strata- e.g. CC, TC and TT GSK3β rs6438552 genotypes were coded as 0, 1 and 2 respectively.

### Statistical analysis

Maximum NCI score, final sural nerve conduction amplitude and i90 amplitude at week 4 were selected as markers of neurotoxicity. Linear regression was utilised for continuous variables and Fisher’s exact test for categorical variables. The effects of polymorphisms on sural amplitude and i90 were examined by linear regression, with paclitaxel dose as an a priori predictor variable. The effects of polymorphisms on maximal NCI score was examined by Fisher’s exact tests. Wilcoxon signed-ranks test was used to compare baseline and final/week4 results for sural and i90 respectively. The two-sided significance level was P ≤ 0.05. As an exploratory analysis, no corrections were done for multiple statistical testing, which should be considered when interpreting the results. All analyses were performed using SPSS (version 21, IBM, NY USA).

## Results

### Clinical details

Clinical details of the 21 paclitaxel-treated patients recruited for the pilot study are shown in Table 1. The majority of patients had breast cancer (95.2%; Stages 1–3A) and received four cycles of doxorubicin 60 mg/m^2^ and cyclophosphamide 600 mg/m^2^ followed by paclitaxel 80 mg/m^2^ weekly for 12 weeks (N = 16). The remaining patients received paclitaxel at 2 or 3 weekly intervals. 76% of patients completed paclitaxel treatment as intended, 14% ceased prematurely due to neurotoxicity, 5% due to disease progression and 5% due to other toxicity.

**Table 1:** **Clinical details**

Clinical variables	
Age (mean ± sem; years)	51.4 ± 2.1
Range (years)	33 – 68
% Female	95.2%
Cumulative paclitaxel dose (mg/m^2^)	965.5 ± 80
Range (mg/m^2^)	420 - 2230
Estrogen and/progesterone receptor positive	80%
HER-2/*neu* positive	40%
Cancer type – breast cancer	95.2%
Cancer stage	
I - IIB	47.6%
IIIA - IIIB	47.6%
IV	4.8%

### Neuropathy assessment

Overall, 76% of patients experienced neuropathic symptoms at any stage, with 56% having a maximum grade of mild (NCI grade 1), 31% moderate (NCI grade 2) and 13% severe (NCI grade 3). Patients underwent clinical and neurophysiological testing at a median of 90 days (range 22 – 378 days) following completion of treatment. Of these, 67% had reduced or absent ankle reflex, 44% had deficits in vibration sense and 28% in pinprick sensibility. Overall, the total neuropathy score was 0–1 in 39%, 2–4 in 50% and greater than 5 in 11%.

43% of patients reported persisting tingling and numbness in the hands and 48% reported persisting symptoms in the feet. 24% of patients reported continuing functional problems with fine motor or walking skills. EORTC CIPN20 questionnaire score was significantly correlated to the maximal NCI grade (correlation coefficient = 0.769; P ≤ .005). Sural amplitude was significantly decreased from baseline pre-treatment to completion of treatment (pre 20.4 ± 2.5 μV; post 14.9 ± 2.3 μV; P ≤ 0.05). i90 was significantly increased by the 4th week of treatment (N = 15; pre 4.56 ± 0.29 mA; week 4 5.54 ± 0.52 mA, P ≤ .01), as in previous studies [15].

### Polymorphism analysis

The proportion of *GSK3B* rs6438552 genotypes was significantly different between patients with no or mild neurotoxicity (Grade 0/1) and those with moderate/severe neurotoxicity (>Grade 2; Fisher’s exact test = 6.411; P ≤ .05), with the T/T genotype associated with reduced neurotoxicity severity (Table 2). However, there were no differences in *MAPT* haplotype or *MAPT* polymorphism rs242557 compared to neurotoxicity grade (Fisher’s exact test = 0.863; NS; Fisher’s exact test = 2.984; NS). Further, patients with the C/C genotype of the GSK3β rs6438552 polymorphism demonstrated an odds ratio of 2 with a 95% CI of .899 to 4.452 with respect to the development of moderate/severe neurotoxicity (P ≤ .05). Accordingly, patients with the T/T polymorphic *GSK3B* alleles did not demonstrate neurotoxicity greater than Grade 1, while patients with the C/C genotype developed only moderate or severe (Grade 2/3) neurotoxicity. Linear regression analysis between polymorphisms and sural amplitude or i90 week 4, with a priori covariate cumulative paclitaxel dose, was not significant for either *GSK3B* rs6438552, *MAPT* rs242557 or *MAPT* haplotype.

**Table 2:** **Analysis of polymorphism status and maximum neurotoxicity grade**

Genotype (N = 21)	NCI grade 0/1	NCI grade 2/3
MAPT haplotype		
11	67%	67%
12	25%	33%
22	8%	0%
MAPT rs24255		
11	42%	17%
12	42%	33%
22	16%	50%
GSK3B rs6438552		
CC	0%	50%
TC	75%	50%
TT	25%	0

## Discussion

The present study examined the potential effects of *MAPT* haplotype, expression levels, and *GSK-3*β mediated tau phosphorylation on the development of paclitaxel-induced neuropathy *in vivo*. Clinical, neurophysiological and genomic approaches, combined with patient questionnaires identified that *GSK3B* polymorphisms may influence the severity of paclitaxel-induced neurotoxicity, with the C/C genotype of the rs6438552 polymorphism associated with greater severity of neurotoxicity. In contrast, there were no identified effects of *MAPT* haplotype or the rs242557 polymorphism on neurotoxicity.

A number of genetic polymorphisms have been previously examined with regards to paclitaxel-induced neurotoxicity, including in drug detoxification pathways [16] and DNA repair mechanisms [17]. The most commonly associated candidate polymorphisms have been in the genes *ABCB1* and *CYP2C8,* associated with the drug metabolism and transport pathways [16, 18–20]. Further, a two-fold increase in severe neurotoxicity with paclitaxel treatment has been identified in patients with polymorphisms in FANCD2, associated with defective DNA repair in the FA/BRCA pathway [17]. Genome-wide association studies in paclitaxel-treated patients have identified some additional associations, but these remain to be confirmed [18, 21, 22]. Such approaches have indicated that polymorphisms in ephrin type A receptors involved in neural injury and repair may be associated with risk of paclitaxel-induced neurotoxicity [23]. However, genetic contributions to paclitaxel-induced neurotoxicity seem likely to be polygenic, with different genes producing multiplier effects on overall neurotoxicity risk [21, 24]. A suite of 4 polymorphisms associated with drug receptors, transcription, apoptosis, and pain perception lead to a cumulative increased neurotoxicity risk of 62% [24].

A recent study of 1303 patients investigated polymorphisms in 50 genes to address this variability, identifying that polymorphisms in the P-glycoprotein transporter gene *ABCB1* and β-tubulin gene *TUBB2A* were the most significantly associated with paclitaxel-induced neurotoxicity, suggesting the role of the tubulin pathway in the pathogenesis of paclitaxel-induced neuropathy [18]. Further, *TUBB2A* polymorphisms were identified in a prior study as protective against paclitaxel neurotoxicity, and found to increase transcription [25]. Similarly, β-tubulin isotype and tau tumour expression are associated with clinical response to paclitaxel [9, 26]. Accordingly, β-tubulin/tau pathway may be relevant in determining toxicity risk and response to paclitaxel treatment.

The H1 haplotype and *MAPT* polymorphism rs242557 increase *MAPT* transcription [6, 27]. Further, the polymorphism rs6438552 in *GSK3β* has functional splicing consequences, with the T allele leading to increased transcription of the splice isoform lacking exons 9 and 11 [22]. The absence of these exonic sequences in *GSK3B* increases the ability to phosphorylate tau, so there is a 3.8 fold increase in tau phosphorylation in the T/T variant compared to the C/C isoform [28].

The present study identified that the T allele of the *GSK3B* polymorphism rs6438552 may be protective against paclitaxel-induced neurotoxicity, with no patients with the homozygous T allele demonstrating neurotoxicity greater than grade 1. However, there were no identified effects of *MAPT* haplotype or the polymorphism rs242557. As discussed, the *GSK3B* rs6438552 T/T polymorphism increases tau phosphorylation [28], which reduces microtubule stability and the proportion of tau associated with microtubules [10]. Further, *GSK3β* mediated phosphorylation of MAPT may be protective against axonal damage *in vivo* [29], suggesting a rationale for risk reduction. Transgenic mice models overexpressing *MAPT* developed anterograde axonal transport deficits and axonopathy in central axons due to excess tau which stabilised microtubules [29]. However, these effects could be ‘rescued’ by phosphorylation from excess *GSK3β*, mediated potentially via protection of axonal transport.

While in neurodegenerative disease *GSK3β*-mediated phosphorylation of tau results in neuronal degradation [30], in paclitaxel-induced neurotoxicity, a reduction in microtubule stabilization may be beneficial to axonal integrity. Both tau and paclitaxel produce enhanced stability of microtubules, interfere with axonal transport, and may share a binding site on *β* –tubulin [13]. Microtubules are dynamic structures and small changes in microtubule organization have major impacts on function and on axonal transport [2]. While the present study identified that polymorphisms in *GSK3B* may be associated with paclitaxel-induced neurotoxicity, a limitation relates to sample size. As such, these findings are preliminary and require confirmation in a larger series. However, an advantage was that objective neurophysiological data was obtained to demonstrate neuropathy, while prior studies utilized clinician-based scales.

## Conclusions

While further studies in a larger cohort will be required to confirm these results, polymorphisms in tau-associated genes (*GSK-3B* rs6438552) may contribute to the development of paclitaxel-induced neurotoxicity. However, it is important that appropriate clinical, patient-focused and objective neurophysiological outcomes are collected. Paclitaxel-induced neurotoxicity remains a prominent complication of treatment, resulting in early treatment discontinuation and impacts on quality of life. The inter-individual variability in the onset and severity of paclitaxel-induced neurotoxicity suggests that patient-specific factors such as genetic polymorphisms may be important in identification of at-risk patients.
